# Cerebrospinal Fluid (CSF) 25-Hydroxyvitamin D Concentration and CSF Acetylcholinesterase Activity Are Reduced in Patients with Alzheimer's Disease

**DOI:** 10.1371/journal.pone.0081989

**Published:** 2013-11-29

**Authors:** Per Johansson, Erik G. Almqvist, Jan-Ove Johansson, Niklas Mattsson, Ulf Andreasson, Oskar Hansson, Anders Wallin, Kaj Blennow, Henrik Zetterberg, Johan Svensson

**Affiliations:** 1 Department of Neuropsychiatry, Skaraborg Central Hospital, Falköping, Sweden; 2 Department of Internal Medicine, Sahlgrenska Academy at the University of Gothenburg, Göteborg, Sweden; 3 Department of Endocrinology, Skaraborg Central Hospital, Skövde, Sweden; 4 Institute of Neuroscience and Physiology, Department of Psychiatry and Neurochemistry, the Sahlgrenska Academy at the University of Gothenburg, Mölndal, Sweden; 5 Center for Imaging of Neurodegenerative Diseases, VA Medical Center, University of California San Francisco, San Francisco, California, United States of America; 6 Clinical Memory Research Unit, Department of Clinical Sciences Malmö, Lund University, Malmö, Sweden; 7 UCL Institute of Neurology, Queen Square, London, United Kingdom; University of Manchester, United Kingdom

## Abstract

**Background:**

Little is known of vitamin D concentration in cerebrospinal fluid (CSF) in Alzheimer´s disease (AD) and its relation with CSF acetylcholinesterase (AChE) activity, a marker of cholinergic function.

**Methods:**

A cross-sectional study of 52 consecutive patients under primary evaluation of cognitive impairment and 17 healthy controls. The patients had AD dementia or mild cognitive impairment (MCI) diagnosed with AD dementia upon follow-up (n = 28), other dementias (n = 12), and stable MCI (SMCI, n = 12). We determined serum and CSF concentrations of calcium, parathyroid hormone (PTH), 25-hydroxyvitamin D (25OHD), and CSF activities of AChE and butyrylcholinesterase (BuChE).

**Findings:**

CSF 25OHD level was reduced in AD patients (*P* < 0.05), and CSF AChE activity was decreased both in patients with AD (*P* < 0.05) and other dementias (*P* < 0.01) compared to healthy controls. None of the measured variables differed between BuChE K-variant genotypes whereas the participants that were homozygous in terms of the apolipoprotein E (APOE) ε4 allele had decreased CSF AChE activity compared to subjects lacking the APOE ε4 allele (*P* = 0.01). In AD patients (n=28), CSF AChE activity correlated positively with CSF levels of total tau (T-tau) (r = 0.44, *P* < 0.05) and phosphorylated tau protein (P-tau) (r = 0.50, *P* < 0.01), but CSF activities of AChE or BuChE did not correlate with serum or CSF levels of 25OHD.

**Conclusions:**

In this pilot study, both CSF 25OHD level and CSF AChE activity were reduced in AD patients. However, the lack of correlations between 25OHD levels and CSF activities of AChE or BuChE might suggest different mechanisms of action, which could have implications for treatment trials.

## Introduction

Calcium influx into cells is a mediator of cellular metabolism, but unbuffered intracellular calcium could be neurotoxic for brain cells [1]. Circulating calcium is dependent on parathyroid hormone (PTH) and vitamin D [2]. In human Alzheimer’s disease (AD), relatively little is known of possible interactions between the calcium/vitamin D system and the cholinergic system. 

The vitamin D receptor (VDR) is abundantly expressed in the human brain [3]. In patients with AD, VDR expression was reduced in hippocampal cells and correlated with calcium binding protein (calbindin-28k) expression [4]. In experimental studies, vitamin D exerted neuroprotective actions by downregulating calcium ion channels [5], and developmental deficiency of vitamin D causes abnormal brain development [6]. Furthermore, 1,25-dihydroxyvitamin D can decrease the activity of reactive oxygen species (ROS) [6], and the defective phagocytosis of soluble amyloid-β (Aβ) by macrophages in AD might be stimulated by vitamin D [7].

VDR polymorphisms or deficiency of vitamin D could be risk factors for cognitive decline as well as AD [8,9]. In systematic reviews/meta-analyses, lower circulating vitamin D concentrations were associated with poorer cognitive function and a higher risk of AD [10,11]. Little is, however, known of intrathecal levels of vitamin D and calcium in relation to cognitive function. No active transport of 25-hydroxyvitamin D (25OHD) into the brain has been observed [6], and under normal conditions, the transport into the brain is relatively restricted [12]. In the brain, there is conversion of 25OHD to 1,25-dihydroxyvitamin D [3].

There is a loss of central cholinergic neurons in severe AD [13]. In early AD, there is no major cholinergic neurodegeneration whereas cholinergic function is reduced, possibly due to imbalances in nerve growth factor expression and changes in the release and receptor expression of acetylcholine [14]. The activity of acetylcholinesterase (AChE), which inactivates cholinergic neurotransmission, is decreased in amygdala, hippocampus and temporal cortex in the AD brain [15]. However, AChE colocalizes with the amyloid core of mature plaques and pre-amyloid diffuse deposits [16]. Aβ-AChE complexes enhance the deregulation of intracellular calcium as well as mitochondrial dysfunction in hippocampal neurons, triggering a more severe damage than Aβ alone [17]. Infusion of AChE into rat hippocampus produced novel plaque-like structures and behavioral deficits developed [18]. Butyrylcholinesterase (BuChE) shares many structural and physic-chemical properties with AChE, but in contrast to AChE, BuChE is increased in the AD brain especially in hippocampus and temporal cortex [15]. BuChE has not been associated with the assembly of Aβ into amyloid fibrils [16], but BuChE might act as a molecular chaperone, thereby suppressing Aβ fibril formation by stabilizing soluble Aβ assemblies [16]. 

Cholinesterase inhibitors are, with moderate effect, used to treat AD. The extent to which treatment with vitamin D is of additional value in this patient group remains to be established. In this study, we determined calcium and vitamin D status in serum and CSF as well as the relations with CSF activities of AChE and BuChE in a well characterized mono-center cohort of patients with cognitive impairment and in matched healthy controls. We also studied whether there were associations with CSF levels of AD biomarkers. 

## Materials and Methods

### Study participants

The study participants as well as AD CSF biomarkers have been reported previously [19]. The study consisted of consecutively recruited Caucasian patients admitted by their general practitioner for primary evaluation of cognitive impairment to a memory clinic in Falköping, Sweden. The participants were recruited by a single specialized physician (P.J.) 2000-2008. Inclusion criteria, beside being referred to Falköping Hospital for evaluation of suspected dementia, were age 65-80 years, body mass index (BMI) 20-26 kg/m^2^, and waist:hip ratio 0.65-0.90 in women and 0.70-0.95 in men. Exclusion criteria were serum creatinine > 175 mmol/L, diabetes mellitus, previous myocardial infarction, malignancy including brain tumor, subdural hematoma, ongoing alcohol abuse, medication with steroids, and previous or present medication with AChE inhibitors. 

Control subjects without any subjective symptoms of cognitive dysfunction were recruited contemporaneously from the same geographical area among spouses of the included patients and by advertisements in local newspapers. Totally, 60 patients (30 men and 30 women) and 20 healthy controls (10 men and 10 women) were recruited. However, in this analysis, 11 subjects (AD, n=4; other dementias, n=3; SMCI, n=1; controls, n=3) receiving medication with calcium or vitamin D were excluded. Therefore, 52 patients (29 men and 23 women) and 17 controls (9 men and 8 women) were included in the present study.

The patients and the control were recruited at various times of the year. AD patients [winter (December - February), spring (March - May), summer (June-August), autumn (September - November); n (%)]: 6 (21%), 9 (32%), 8 (29%), 5 (18%); patients with other dementias: 5 (42%), 2 (17%), 3 (25%), 2 (17%); SMCI patients: 5 (42%), 2 (17%), 0 (0%), 5 (42%); healthy controls: 5 (29%), 9 (53%), 1 (6%), 2 (12%). Thus, although there were no statistically significant difference between the study groups (chi-square *P* - value = 0.09), there were non-significant tendencies that a higher number of patients with other dementias had been recruited during winter and that SMCI patients had been recruited mainly during winter and autumn.

The presence or absence of dementia was diagnosed according to the Diagnostic and Statistical Manual of Mental Disorders, Fourth Edition (DSM-IV), criteria. Patients with dementia were classified as suffering of AD [20], vascular dementia (VaD) according to the requirements by NINDS-AIREN [21] or the guidelines by Erkinjuntti et al. for the subcortical type of VaD [22]. Frontotemporal dementia, Parkinson disease dementia, and dementia with Lewy bodies were diagnosed as described previously [19]. 

Mild cognitive impairment (MCI) was diagnosed in patients with cognitive impairment that did not fulfill the criteria for dementia [23]. Patients with MCI were followed at least annually for a median of 3 (range 1-7) years to evaluate whether they later developed dementia. The causes of the cognitive impairment in the included patients are given in Table 1. All diagnoses were assessed by an independent specialized physician (O.H.). At primary evaluation, 5 of the 28 AD patients had signs of additional vascular pathology according to brain imaging, but these patients did not differ from the remaining AD patients in terms of CSF levels of the AD biomarkers β-amyloid_1-42_ (Aβ_1-42_), total-tau (T-tau) or phosphorylated tau protein (P-tau) (data not shown). During the follow-up visits, 12 MCI patients remained in stable cognitive function (SMCI). Others progressed, during the follow-up period, to dementia and were diagnosed with AD (n = 6) or VaD (n = 3). MCI patients diagnosed with AD on follow-up visits did not differ in CSF levels of Aβ_1-42_, T-tau and P-tau from patients with established AD at baseline (data not shown). Totally, the study population consisted of AD dementia or MCI diagnosed with AD dementia upon follow-up (n = 28), other dementias (n = 12), SMCI (n = 12), and healthy controls (n = 17) (Table 1).

**Table 1:** Diagnoses in 52 patients with cognitive impairment not receiving calcium or vitamin D therapy.

**Diagnosis**	**Number**
All AD	28 (54%)
MCI-AD	6 (12%)
AD	22 (42%)
Other dementias	12 (23%)
MCI-VaD	3 (6%)
VaD	6 (11%)
DLB	3 (6%)
SMCI	12 (23%)

All diagnoses were assessed by an independent specialized physician.

MCI-AD, mild cognitive impairment (MCI) that later converted to Alzheimer’s disease (AD); MCI-VaD, MCI that later converted to vascular dementia (VaD); DLB, dementia with Lewy bodies; SMCI, stable mild cognitive impairment.

### Ethical considerations

The study was approved by the ethical committee of Göteborg University. Ethical approval number: S 496-99. The specialist physician (P.J.) gave written information of the study and explained the study protocol to the controls as well as to the patients and caretaker if available. All participants provided both oral and written informed consent. A next of kin, caretaker or guardian consented on behalf of patients if the capacity to consent was compromised. However, in all cases, the patient's own opinion was asked and considered, and the patient was recruited in the study only when he or she agreed with this. The ethical committee approved this informed consent procedure. The study was conducted according to the principles in the Declaration of Helsinki.

### Cognitive and physical examination

Before the test day, a mini-mental state examination (MMSE) [24] was performed. On the test day morning with the patients in the fasted state, before lumbar puncture was performed, body weight, body height, body mass index (BMI), waist circumference, and hip girth were determined according to standard procedures described previously [19].

### CSF sampling

All CSF samples were collected by lumbar puncture in the L3/L4 or L4/L5 interspace at the standardized time point 08:30-09:00 h. The first 12 mL of CSF was collected in a polypropylene tube and immediately transported to the local laboratory for centrifugation at 2.000g at +4°C for 10 minutes. The supernatant was pipetted off, gently mixed to avoid possible gradient effects, and aliquoted in polypropylene tubes that were stored at -80 °C pending biochemical analyses, without being thawed and re-frozen. 

### Blood samples

CSF and blood samples were obtained at the same visit. Blood samples were drawn in the morning in the fasted state and serum was prepared by centrifugation after coagulation at room temperature for 15-30 min, aliquoted and stored in cryotubes at -80 °C pending biochemical analyses, without being thawed and re-frozen.

### Biochemical procedures and genetic analyses

All biochemical analyses were performed with the analyst blinded to the clinical diagnoses and other clinical information. All analyses were performed at one occasion. 

Total calcium concentrations in serum and CSF were measured using the o-cresolphthalein method on a Roche Cobas c501 instrument (Roche Diagnostics, Penzberg, Germany). Serum and CSF levels of total 25OHD were analyzed using the Vitamin D Total immunoassay on a Roche Cobas e601 instrument (Roche Diagnostics). Serum levels of PTH were measured using an intact PTH immunoassay of sandwich type on an ARCHITECT i2000 instrument (Abbott Diagnostics, Abbott Park, IL, USA). CSF concentrations of PTH were below the lower detection limit of the assay (3 ng/L). CSF AChE and BuChE activities were determined using in house assays as previously described in detail [25].

CSF biomarkers were measured at the Clinical Neurochemistry Laboratory in Mölndal, Sweden, by experienced laboratory technicians. CSF Aβ_1-42_ levels were determined using the INNOTEST® ELISA assay technology (Innogenetics, Ghent, Belgium) [26]. The axonal damage marker CSF T-tau and CSF concentrations of tau phosphorylated at threonine 181 (P-tau181) were measured using INNOTEST® ELISA assays [27,28].

Apolipoprotein E (APOE) genotyping was performed by minisequencing as described previously in detail [29]. Genotypes were obtained for the two SNPs, which are used to unambiguously define ε2, ε3, and ε4 alleles (rs7412 and rs429358). Genotyping of the BuChE K-variant (rs1803274) was performed using TaqMan® Allelic Discrimination technology (Applied Biosystems, Foster City, CA, USA) [30].

### Statistical analyses

Statistical analyses were performed using SPSS for Windows (version 17.0; SPSS, Chicago, IL, USA). The descriptive statistical results are given as the median (25th-75th percentile) if not otherwise stated. Between-group differences for continuous variables were assessed using the non-parametric Kruskal-Wallis test for multiple variables, followed by the Mann-Whitney U test for pair-wise comparisons. Differences between groups for categorical variables were assessed using chi-square tests. Correlations were sought using the Spearman rank order correlation test. Significance was obtained if the two-tailed *P*-value was ≤ 0.05. 

## Results

The patients and the controls were comparable in terms of age, gender, BMI, and waist:hip ratio (Table 2). A more detailed clinical description as well as CSF AD biomarkers for the original cohort have been reported previously [19]; data for the subjects included in this study are shown in Table 2. None of the investigated CSF biomarkers correlated with age or albumin ratio in the whole population or in any study group (*P* > 0.05).

**Table 2:** Age, anthropometric measures, MMSE score and CSF biomarkers in the study population of 52 patients with cognitive impairment (AD, n=28; other dementias, n=12; SMCI, n=12) and 17 healthy matched controls.

	**AD**	**Other dementias**	**SMCI**	**Controls**	P-value*
**Men/women**	14/14	10/2	5/7	9/8	0.17
**Age (years)**	74 (71-77)	74 (72-76)	72 (69-73)	75 (70-78)	0.19
**BMI (kg/m^2^)**	23.4 (21.8-25.6)	22.4 (20.6-25.3)	24.8 (23.0-26.0)	23.7 (23.0-25.1)	0.30
**Waist/hip ratio**	0.87 (0.82-0.91)	0.91 (0.89-0.94)	0.84 (0.78-0.89)	0.87 (0.82-0.91)	0.33
**MMSE score**	23 (18-25)***^a^***,***^b^***	23 (19-25)***^a^***,***^c^***	29 (28-29)	29 (27-29)	< 0.0001
**Aβ_1-42_ (ng/L)**	420 (339-486)***^a^***,***^c^***	393 (338-638)***^d^***,***^e^***	746 (552-868)**^*f*^**	993 (870-1045)	< 0.0001
**T-tau (ng/L)**	600 (459-810)**^a,b,g^**	303 (215-366)	276 (230-396)	322 (224-369)	< 0.0001
**P-tau (ng/L)**	105 (80-119)**^c,d,g^**	46 (32-60)**^*h*^**	59 (38-78)	64 (51-80)	< 0.0001

* P-values for differences between all groups were assessed using the Kruskal-Wallis test for multiple variables. Post hoc testing was then performed using the Mann-Whitney U test for pair-wise comparisons.

Values are given as the median (25th-75th percentile).

aP < 0.0001 vs. controls; bP < 0.0001 vs. SMCI; cP < 0.001 vs. SMCI; dP < 0.001 vs. controls; eP = 0.04 vs. SMCI; fP = 0.006 vs. controls; gP < 0.0001 vs. other dementias; hP = 0.01 vs. controls.

### Calcium and vitamin D in serum and CSF

Serum levels of total calcium, PTH, and 25OHD did not differ significantly between study groups (Table 3). In CSF, PTH was not measurable. CSF 25OHD level was lower in AD patients compared to patients with other dementias and healthy controls (*P* = 0.01 and *P* = 0.03, respectively; Table 3). CSF total calcium level was similar in all study groups. There were no between-group differences in terms of CSF/serum ratios of calcium or 25OHD. 

**Table 3:** Serum and CSF levels of calcium, PTH, and 25-hydroxyvitamin D (25OHD) in the study population of 52 patients with cognitive impairment (AD, n=28; other dementias, n=12; SMCI, n=12) and 17 healthy matched controls.

	**AD**	**Other dementias**	**SMCI**	**Controls**	P-value*
**Serum levels**					
Calcium (mmol/L)	2.37 (2.30-2.45)	2.39 (2.33-2.45)	2.35 (2.32-2.43)	2.37 (2.35-2.39)	0.92
PTH (ng/L)	54.5 (46.5-71.5)	54.0 (38.0-56.5)	54.0 (46.5-74.0)	45.0 (41.5-58.8)	0.15
25OHD (nmol/L)	44.0 (36.0-54.5)	42.0 (37.0-49.0)	41.0 (37.8-55.8)	56.0 (46.8-57.3)	0.31
**CSF levels**					
25OHD (nmol/L)	11.0 (10.0-11.5)***^a^***,***^b^***	12.0 (11.5-12.5)	11.0 (10.0-12.0)	12.0 (11.0-13.0)	0.03
Calcium (mmol/L)	1.16 (1.14-1.20)	1.15 (1.11-1.19)	1.19 (1.18-1.23)	1.19 (1.15-1.20)	0.16
**Ratios**					
CSF/serum 25OHD	0.23 (0.19-0.28)	0.28 (0.23-0.32)	0.26 (0.19-0.27)	0.22 (0.21-0.23)	0.13
CSF/serum calcium	0.49 (0.48-0.50)	0.48 (0.47-050)	0.50 (0.49-0.51)	0.50 (0.49-0.51)	0.09

PTH was only measurable in serum.

* P-values for differences between all groups were assessed using the Kruskal-Wallis test for multiple variables. Post hoc testing was then performed using the Mann-Whitney U test for pair-wise comparisons.

Values are given as the median (25th-75th percentile).

***^a^*** *P* = 0.03 *vs*. controls; ***^b^*** *P* = 0.01 *vs*. other dementias

### AChE and BuChE activities in CSF

**Figure 1:**
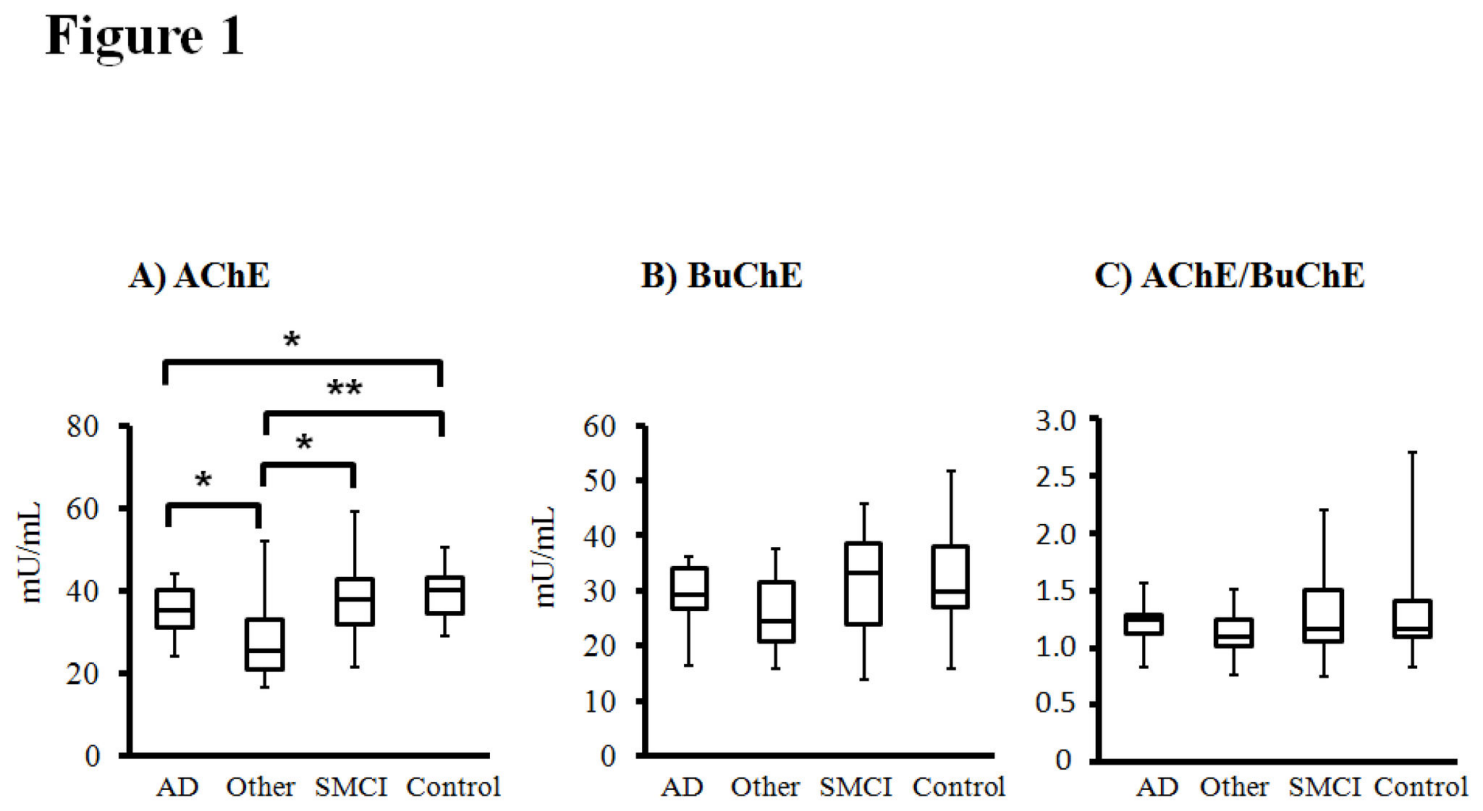
Reduced CSF acetylcholinesterase (AChE) activity both in patients with AD and other dementias compared to healthy controls. A) CSF AChE activity, B) CSF butyrylcholinesterase (BuChE) activity, and C) CSF AChE/BuChE ratio in the study population of patients with AD (n = 28), other dementias (n = 12), SMCI (n = 12), and healthy controls (n = 17). Values in the box plots are given as medians (horizontal lines), 25th-75th percentiles (boxes), and ranges (whiskers). Between-group differences were assessed using the Kruskal-Wallis test for multiple variables, followed by the Mann-Whitney U test for pair-wise comparisons. ^*^ *P* < 0.05 ^**^ *P* < 0.01 .

AChE activity in CSF was lower in AD patients compared with controls (*P* = 0.049) (Figure 1A). In addition, CSF AChE activity was lower in patients with other dementias than that in patients with AD, SMCI or controls (*P* = 0.01, *P* = 0.01 and *P* = 0.002, respectively) (Figure 1A). BuChE activity in CSF as well as the ratio between AChE and BuChE activities in CSF was similar in all study groups (Figure 1B and 1C). 

### Correlation analysis

Serum and CSF levels of 25OHD correlated positively both in the total study population (n = 69; r = 0.39, *P* = 0.002) and in AD patients (n = 28; r = 0.39, *P* = 0.04). Serum and CSF values of total calcium correlated positively in the total study population (r = 0.37, *P* = 0.002) but not significantly in the AD group (r = 0.35, P = 0.07). The only observed correlations between the calcium/vitamin D system and CSF activities of AChE and BuChE were the positive correlations between CSF total calcium and AChE (r = 0.30, *P* = 0.01) and BuChE (r = 0.32, *P* = 0.01) in the total study population.

**Figure 2:**
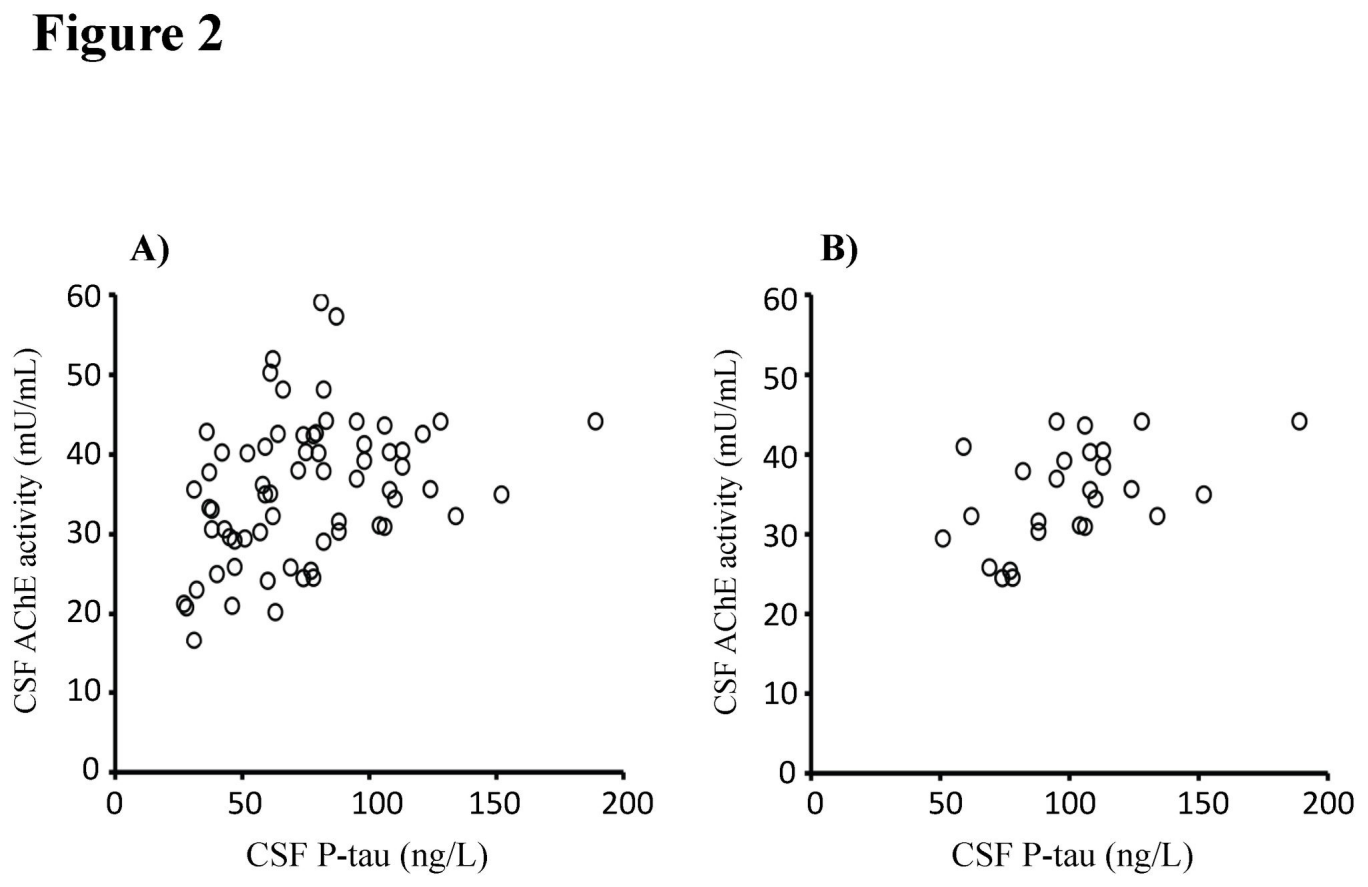
CSF acetylcholinesterase (AChE) activity correlates positively with the CSF level of the AD biomarker phosphorylated tau protein (P-tau). A) In the total study population (n = 69; r = 0.41, *P* < 0.001) as well as in B) AD patients (n = 28; r = 0.50, *P* = 0.009), CSF AChE activity correlated positively with CSF P-tau level. Correlations were sought using the Spearman rank order correlation test.

We then investigated whether variables that significantly differed between the study groups (CSF 25OHD level and CSF AChE activity) correlated with CSF AD biomarkers. In the total study population (n = 69), CSF AChE activity correlated positively with Aβ_1-42_ (r = 0.47, *P* < 0.001) and P-tau (r = 0.41, *P* < 0.001; Figure 2A). In separate analyses of the AD patients (n = 28), CSF AChE activity correlated positively with T-tau (r = 0.44, *P* = 0.02) and P-tau (r = 0.50, *P* = 0.009; Figure 2B). CSF 25OHD level did not correlate with CSF AD biomarkers in the total study population or in AD patients (data not shown). 

In the total study population (n=69), BMI correlated positively with CSF activities of BuChE (r = 0.34, *P* = 0.006) and AChE (r = 0.26, *P* = 0.04). In AD patients analyzed separately (n=28), BMI correlated positively with CSF BuChE activity (r = 0.51, *P* = 0.01) but not with CSF AChE activity (r = 0.28, *P* = 0.17). BMI did not correlate with any of the variables reflecting calcium/vitamin D metabolism or with CSF AD biomarkers (data not shown).

### Genetic influence

The distribution of the BuChE K-variant was similar in the different study groups whereas AD patients had higher frequency of the APOE ε4 allele than the other study groups (*P* = 0.03, Table 4). CSF activities of AChE or BuChE did not differ between BuChE K-variant genotypes whereas the participants that were homozygous in terms of the APOE ε4 allele had decreased CSF AChE activity compared to subjects lacking the APOE ε4 allele (*P* = 0.01; Table 5). Also after exclusion of AD patients, CSF AChE activity was lower in participants that were homozygous in terms of the APOE ε4 allele compared to subjects lacking the APOE ε4 allele [median (25th -75th percentile) in homozygous patients: 23.1 (17.8-25.7) mU/mL vs. in patients lacking APOE ε4 allele: 40.3 (31.8-43.5) mU/mL, *P* = 0.002].

**Table 4:** The distribution of BuChE K and APOE genotypes in the study population of 52 patients with cognitive impairment (AD, n=28; other dementias, n=12; SMCI, n=12) and 17 healthy matched controls.

	**AD**	**Other dementias**	**SMCI**	**Controls**	P-value*
BuChE K allele (--/-+/++)	21 / 6 / 1 (75% / 21% / 4%)	8 / 4 / 0 (67% / 33% / 0%)	8 / 3 / 1 (67% / 25% / 8%)	6 / 8 / 3 (35% / 47% / 18%)	0.16
APOE ε4 allele (--/-+/++)	9 / 11 / 7 **^*a*^** (33% / 41% / 26%)	5 / 3 / 3 (46% / 27% / 27%)	6 / 4 / 1 (55% / 36% / 9%)	14 / 3 / 0 (82% / 18% / 0%)	0.04
≥ 1 BuChE K allele and ≥ 1 APOE ε4 allele (yes/no)	4 (14%) / 24 (86%)	2 (17%) / 10 (83%)	1 (8%) / 11 (92%)	2 (12%) / 15 (88%)	0.93

* Differences between groups were assessed using chi-square tests.

Values are given as number (%). APOE genotyping was not performed in three of the study participants.

*a* *P* = 0.03 *vs*. controls

**Table 5:** CSF activities of AChE and BuChE in relation to BuChE K-variant genotype or APOE ε4 allele distribution in the total study population.

**BuChE K-variant**	**None (n=43, 62%)**	**Heterozygous (n=21, 31%)**	**Homozygous (n=5, 7%)**	P-value*
AChE (mU/mL)	35.3 (29.6-42.4)	38.5 (29.9-42.6)	33.1 (29.4-36.3)	0.68
BuChE (mU/mL)	30.5 (26.4-34.7)	27.4 (22.2-36.0)	27.6 (18.8-28.8)	0.22
**APOE ε4 allele**	**None (n=34, 51%)**	**Heterozygous (n=21, 32%)**	**Homozygous (n=11, 17%)**	P-value*
AChE (mU/mL)	38.0 (30.9-42.9)	35.6 (29.4-40.2)	25.9 (24.5-37.5)**^*a*^**	0.03
BuChE (mU/mL)	29.4 (25.4-36.2)	28.7 (25.9-34.2)	27.5 (24.9-30.0)	0.39

* P-values for differences between all groups were assessed using the Kruskal-Wallis test for multiple variables. Post hoc testing was then performed using the Mann-Whitney U test for pair-wise comparisons.

Values are given as the median (25th-75th percentile). APOE genotyping was not performed in three of the study participants.

*a* *P* = 0.01 *vs*. participants lacking the APOE ε4 allele.

Levels of calcium, PTH, and 25OHD were not dependent on the distribution of the BuChE K-variant or the APOE ε4 allele (data not shown). 

### Subanalyses of gender differences

In the total study population (n=69), we investigated whether levels of the measured variables differed between men and women. Only serum 25OHD level differed between genders with lower values in women [median (25th -75th percentile): 40.5 (34.3-53.5) nmol/L] compared to men [52.0 (41.0-56.3) nmol/L, *P* = 0.03]. We then performed gender-specific analyses using the Kruskal-Wallis test to compare the four study groups with respect to serum 25OHD level. In men analyzed separately, there was no between-group difference (*P* = 0.19) whereas in women, serum 25OHD level differed between the study groups (*P* = 0.01). Post hoc analyses using the Mann-Whitney U test showed lower serum 25OHD level in female AD patients [36.0 (27.0-44.8) nmol/L] than in female SMCI patients [48.0 (38.8-56.5) nmol/L, *P* = 0.03] and female controls [53.0 (46.3-60.3) nmol/L, *P* = 0.004], but otherwise there were no difference between groups in women.

## Discussion

In the present study, CSF 25OHD level was reduced in AD patients, and CSF AChE activity was decreased both in patients with AD and other dementias compared to healthy controls. None of the measured variables differed between BuChE K-variant genotypes whereas the participants that were homozygous in terms of the APOE ε4 allele had decreased CSF AChE activity compared to subjects lacking the APOE ε4 allele. In AD patients (n=28), CSF AChE activity correlated positively with CSF levels of T-tau and P-tau, but CSF activities of AChE or BuChE activity did not correlate with serum or CSF levels of 25OHD.

Little is known of CSF 25OHD concentrations in human AD. In the present study, CSF 25OHD level was lower in AD patients compared to patients with other dementias and healthy controls. As previously observed in healthy individuals [31] and in multiple sclerosis [32], serum and CSF levels of 25OHD correlated positively. There was no between-group difference in terms of ratios between CSF and serum levels of 25OHD, and in AD patients as well as in the controls, the median CSF 25OHD level was one fifth to one fourth of that in the circulation. This likely suggests that the CSF levels reflect those in serum. There was only a non-significant tendency to lower serum 25OHD in the AD group, which could be due to a limited number of patients and therefore possibly low statistical power. However, in AD patients, the median serum 25OHD level was below 50 nmol/L, which has been defined as the upper limit for vitamin D deficiency [33]. In contrast, the median level was above 50 nmol/L in the healthy controls. 

All the included patients were community-dwelling and in the early phases of cognitive impairment/dementia with only a moderate reduction of MMSE score. Non-institutionalized older individuals have better vitamin D status compared to those living in homes for elderly as dietary intake of vitamin D and sun exposure are low in the latter group [33]. In older community-dwellers with subjective memory complaint but without overt dementia, low serum 25OHD level was associated with increased risk of MCI [34]. Our findings concur with these results and suggest that a derangement in vitamin D level exist already in an early phase of cognitive impairment/dementia. However, with increasing duration of disease, vitamin D status may gradually deteriorate, which could accelerate the progression of the dementing disorder. 

In line with the results of a previous study [35], CSF BuChE activity was similar in AD patients and healthy controls. Furthermore, in agreement with the results of some [35] but not all [36] studies, CSF AChE activity was decreased in the AD group compared to the controls. Assessment of AChE activity in CSF can be influenced by several factors such as drug intake, time of day when lumbar puncture was performed, diet and environment, and fraction of CSF assayed [37]. However, the present study was performed under highly standardized procedures regarding lumbar puncture and laboratory assays. Patients and controls were matched in terms of age, gender, BMI, and waist: hip ratio. All patients were included at one center and diagnosed by one physician (with an independent evaluator corroborating the diagnoses). A previous analysis showed a very high diagnostic accuracy of the core AD biomarkers Aβ_1-42_, T-tau, and P-tau [19]. Furthermore, no patient had ever received treatment with an acetylcholinesterase inhibitor and none of the participants received medication with glucocorticoids, calcium or vitamin D. 

CSF AChE activity was lower in the other dementia group compared to all other study groups. Some previous studies have also reported a more marked decrease of CSF AChE activity in VaD than in AD [36,38]. However, our study group with other dementia was heterogeneous with few cases of each specific diagnosis such as VaD, and larger studies than the present one are therefore needed to evaluate whether the degree of downregulation of CSF AChE activity is disease-specific in dementing disorders.

Although this could have been due to limited statistical power, the distribution of the K-variant was similar in all study groups. Furthermore, in the total study population, CSF activities of AChE or BuChE were not dependent on BuChE K-variant distribution. Previous studies have shown conflicting results with an increased risk of late-onset AD in the presence of both the K-variant and the APOE ε4 allele in one study [39] but not in another study [40]. McIlroy et al. reported that possession of the BuChE K allele constituted an increased risk for AD without any synergism with APOE ε4 allele distribution [41] whereas a recent study showed an overrepresentation of the BuChE K-variant in AD with reduced CSF BuChE activity in carriers of both the BuChE K-variant and the APOE ε4 allele [30]. In our study, participants that were homozygous carriers of the APOE ε4 allele had decreased CSF AChE activity compared to subjects lacking the APOE ε4 allele, possibly giving some additional support that APOE ε4 allele distribution could be related to the function of the cholinergic system. We did not, however, observe that any component of the calcium/vitamin D system was dependent on the distribution of the BuChE K-variant or the APOE ε4 allele. 

In a previous study, AD patients treated with AChE inhibitors had higher vitamin D levels than non-treated patients [42]. In our study, CSF total calcium level correlated positively with CSF activities of AChE and BuChE in the total study population. Otherwise, none of the components of the calcium/vitamin D system correlated with CSF AChE or BuChE activity, suggesting that levels of 25OHD, calcium, or PTH are only to a small extent related to CSF activities of AChE or BuChE. Vitamin D might affect cognition at least partly independent of AChE and BuChE involving L-type voltage-sensitive calcium channels, nerve growth factor, prostaglandins, cyclooxygenase 2, reactive oxygen species, nitric oxide synthase, and prevention of secondary hyperparathyroidism [2]. In further support of different mechanisms of action, CSF AChE activity correlated with CSF levels of AD biomarkers in the total study population as well as in AD patients whereas CSF 25OHD level did not. 

Different mechanisms of action in terms of vitamin D and AChE could be of clinical importance. Treatment of AD patients with AChE inhibitors have resulted in limited clinical effects. In terms of vitamin D, systematic reviews/meta-analyses of epidemiological data showed that lower circulating vitamin D concentrations were associated with poorer cognitive function and a higher risk of AD [10,11]. In a memory clinic study with pre-post design without randomization, outpatients that received vitamin D3 supplements had improved cognitive function compared to non-treated outpatients [43]. A six-month pilot study showed that combined treatment with vitamin D and the N-methyl-D-aspartate receptor blocker memantine exerted additive effects on MMSE score compared with either drug alone in early AD [44]. However, the large-scale placebo-controlled studies needed to confirm these results are still lacking. Further studies are therefore warranted to evaluate whether vitamin D treatment, with or without concomitant therapy with an AChE inhibitor, could improve cognitive function in AD patients with marked deficiency of vitamin D. 

One weakness of this study is the cross-sectional design, and changes over time in calcium/vitamin D metabolism and CSF activity of cholinesterases could therefore not be followed. Furthermore, this was a pilot study with a relatively small study population, and the results therefore need to be confirmed in further studies. Gender distribution was statistically similar in the study groups, but it can not be excluded that small differences in gender distribution could have been of importance as subanalyses showed lower serum 25OHD level in female but not in male AD patients compared to gender-matched SMCI patients and controls. In the group with other dementia, there were non-significant tendencies that BMI as well as the proportion of women were lower, and that the proportion of patients recruited during winter were higher, compared to the other study groups. Finally, BMI correlated positively with CSF BuChE activity both in the total study population and in AD patients and in addition, BMI correlated positively with CSF AChE activity in the total study population. Small, non-significant differences between groups in terms of body composition could therefore have influenced our results in terms of CSF activities of BuChE and AChE. 

In conclusion, in a homogenous, well-controlled study cohort, the CSF level of 25OHD was reduced in AD patients compared to patients with other dementias and healthy controls. The reduced CSF AChE activity correlated with CSF levels of AD biomarkers whereas CSF 25OHD level did not. Only CSF AChE activity was dependent on APOE genotype with the participants that were homozygous in terms of the APOE ε4 allele having decreased CSF AChE activity compared to subjects lacking the APOE ε4 allele. Furthermore, there was no correlation between levels of 25OHD and CSF activities of cholinesterases. Therefore, both 25OHD and AChE could be of importance for cognitive function in AD although possibly by different mechanisms of action. These findings might suggest that there is a rationale of combining vitamin D and AChE inhibitors in treatment trials. 

## References

[1] Shetty PK, Galeffi F, Turner DA (2011) Age-induced alterations in hippocampal function and metabolism. Aging DIS 2: 196-218. PubMed: 22081793. 22081793 PMC3212402

[2] Lu'o'ng K, Nguyên L (2011) The beneficial role of vitamin D in Alzheimer's disease. Am; Alzheimers J Dis Other Demen 26: 511-520. 10.1177/1533317511429321 PMC10845314 22202127

[3] Eyles DW, Feron F, Cui X, Kesby JP, Harms LH et al. (2009) Developmental vitamin D deficiency causes abnormal brain development. Psychoneuroendocrinology 34S: S247—S257. PubMed: 19500914. 10.1016/j.psyneuen.2009.04.015 19500914

[4] Sutherland M, Somerville M, Yoong L, Bergeron C, Haussler M et al. (1992) Reduction of vitamin D hormone receptor mRNA levels in Alzheimer as compared to Huntington hippocampus: correlation with calbindin-28k mRNA levels. Brain. Resour - Mol Brain Res 13: 239-250. doi:10.1016/0169-328X(92)90032-7. 1317496

[5] Brewer LD, Thibault V, Chen KC, Langub MC, Landfield PW et al. (2001) Vitamin D hormone confers neuroprotection in parallel with downregulation of L-type calcium channel expression in hippocampal neurons. J Neurosci 21: 98-108. PubMed: 11150325. 11150325 10.1523/JNEUROSCI.21-01-00098.2001 PMC6762438

[6] Eyles DW, Burne TH, McGrath JJ (2013) Vitamin D, effects on brain development, adult brain function and the links between low levels of vitamin D and neuropsychiatric disease. Front Neuroendocrinol 34: 47-64. doi:10.1016/j.yfrne.2012.07.001. PubMed: 22796576. 22796576

[7] Masoumi A, Goldenson B, Ghirmai S, Avagyan H, Zaghi J et al. (2009) 1alpha,25-dihydroxyvitamin D3 interacts with curcuminoids to stimulate amyloid-beta clearance by macrophages of Alzheimer's disease patients. J Alzheimers Dis 17: 703-717. PubMed: 19433889. 19433889 10.3233/JAD-2009-1080

[8] Annweiler C, Schott AM, Allali G, Bridenbaugh SA, Kressig RW et al. (2010) Association of vitamin D deficiency with cognitive impairment in older women: cross-sectional study. Neurology 74: 27-32. doi:10.1212/WNL.0b013e3181d0ccb7. PubMed: 19794127. 19794127

[9] Lehmann DJ, Refsum H, Warden DR, Medway C, Wilcock GK et al. (2011) The vitamin D receptor gene is associated with Alzheimer's disease. Neurosci Lett 504: 79-82. doi:10.1016/j.neulet.2011.08.057. PubMed: 21911036. 21911036

[10] Balion C, Griffith LE, Strifler L, Henderson M, Patterson C et al. (2012) Vitamin D, cognition, and dementia: a systematic review and meta-analysis. Neurology 79: 1397-1405. doi:10.1212/WNL.0b013e31826c197f. PubMed: 23008220. 23008220 PMC3448747

[11] Annweiler C, Llewellyn DJ, Beauchet O (2013) Low serum vitamin D concentrations in Alzheimer's disease: a systematic review and meta-analysis. J Alzheimers Dis 33: 659-674. PubMed: 23042216. 23042216 10.3233/JAD-2012-121432

[12] Pardridge WM, Sakiyama R, Coty WA (1985) Restricted transport of vitamin D and A derivatives through the rat blood-brain barrier. J Neurochem 44: 1138-1141. doi:10.1111/j.1471-4159.1985.tb08735.x. PubMed: 3838342. 3838342

[13] Whitehouse PJ, Price DL, Struble RG, Clark AW, Coyle JT et al. (1982) Alzheimer's disease and senile dementia: loss of neurons in the basal forebrain. Science 215: 1237-1239. doi:10.1126/science.7058341. PubMed: 7058341. 7058341

[14] Schliebs R, Arendt T (2011) The cholinergic system in aging and neuronal degeneration. Behav Brain Res 221: 555-563. doi:10.1016/j.bbr.2010.11.058. PubMed: 21145918. 21145918

[15] Perry EK, Perry RH, Blessed G, Tomlinson BE (1978) Changes in brain cholinesterases in senile dementia of Alzheimer type. Neuropathol Appl Neurobiol 4: 273-277. doi:10.1111/j.1365-2990.1978.tb00545.x. PubMed: 703927. 703927

[16] Inestrosa N, Dinamarca M, Alvarez A (2008) Amyloid-cholinesterase interactions. Implications for Alzheimer'S Disease. FEBS J 275: 625-632. 10.1111/j.1742-4658.2007.06238.x 18205831

[17] Dinamarca MC, Sagal JP, Quintanilla RA, Godoy JA, Arrázola MS et al. (2010) Amyloid-beta-Acetylcholinesterase complexes potentiate neurodegenerative changes induced by the Abeta peptide. Implications for the pathogenesis of Alzheimer's disease. Mol Neurodegener 5: 4. doi:10.1186/1750-1326-5-4. PubMed: 20205793. 20205793 PMC2823746

[18] Chacón MA, Reyes AE, Inestrosa NC (2003) Acetylcholinesterase induces neuronal cell loss, astrocyte hypertrophy and behavioral deficits in mammalian hippocampus. J Neurochem 87: 195-204. doi:10.1046/j.1471-4159.2003.01985.x. PubMed: 12969266. 12969266

[19] Johansson P, Mattsson N, Hansson O, Wallin A, Johansson JO et al. (2011) Cerebrospinal fluid biomarkers for Alzheimer’s disease: diagnostic performance in a homogeneous mono-center population. J Alzheimers Dis 24: 537-546. PubMed: 21297262. 21297262 10.3233/JAD-2011-101878

[20] McKhann G, Drachman D, Folstein M, Katzman R, Price D et al. (1984) Clinical diagnosis of Alzheimer's disease: report of the NINCDS-ADRDA Work Group under the auspices of Department of Health and Human Services Task Force on Alzheimer's Disease. Neurology 34: 939-944. doi:10.1212/WNL.34.7.939. PubMed: 6610841. 6610841

[21] Román G, Tatemichi T, Erkinjuntti T, Cummings J, Masdeu J et al. (1993) Vascular dementia: diagnostic criteria for research studies. Report of the NINDS-AIREN International Workshop. Neurology 43 pp. 250-260. 10.1212/wnl.43.2.250 8094895

[22] Erkinjuntti T, Inzitari D, Pantoni L, Wallin A, Scheltens P et al. (2000) Research criteria for subcortical vascular dementia in clinical trials. Journal of Neural Transm Suppl 59: 23-30. 10.1007/978-3-7091-6781-6_4 10961414

[23] Petersen RC (2004) Mild cognitive impairment as a diagnostic entity. J Intern Med 256: 183-194. doi:10.1111/j.1365-2796.2004.01388.x. PubMed: 15324362. 15324362

[24] Folstein MF, Folstein SE, McHugh PR (1975) "Mini-mental state". A practical method for grading the cognitive state of patients for the clinician. J Psychiatr Res 12: 189-198. doi:10.1016/0022-3956(75)90026-6. PubMed: 1202204. 1202204

[25] Parnetti L, Chiasserini D, Andreasson U, Ohlson M, Hüls C et al. (2011) Changes in CSF acetyl- and butyrylcholinesterase activity after long-term treatment with AChE inhibitors in Alzheimer’s disease. Acta Neurol Scand 124: 122-129. doi:10.1111/j.1600-0404.2010.01435.x. PubMed: 20880294. 20880294

[26] Vanderstichele H, Van Kerschaver E, Hesse C, Davidsson P, Buyse MA et al. (2000) Standardization of measurement of beta-amyloid(1-42) in cerebrospinal fluid and plasma. Amyloid 7: 245-258. doi:10.3109/13506120009146438. PubMed: 11132093. 11132093

[27] Blennow K, Wallin A, Agren H, Spenger C, Siegfried J et al. (1995) Tau protein in cerebrospinal fluid: a biochemical marker for axonal degeneration in Alzheimer disease? Mol Chem Neuropathol 26: 231-245. doi:10.1007/BF02815140. PubMed: 8748926. 8748926

[28] Vanmechelen E, Vanderstichele H, Davidsson P, Van Kerschaver E, Van Der Perre B et al. (2000) Quantification of tau phosphorylated at threonine 181 in human cerebrospinal fluid: a sandwich ELISA with a synthetic phosphopeptide for standardization. Neurosci Lett 285: 49-52. doi:10.1016/S0304-3940(00)01036-3. PubMed: 10788705. 10788705

[29] Blennow K, Ricksten A, Prince JA, Brookes AJ, Emahazion T et al. (2000) No association between the alpha2-macroglobulin (A2M) deletion and Alzheimer's disease, and no change in A2M mRNA, protein, or protein expression. J Neural Transm 107: 1065-1079. doi:10.1007/s007020070052. PubMed: 11041282. 11041282

[30] Darreh-Shori T, Siawesh M, Mousavi M, Andreasen N, Nordberg A (2012) Apolipoprotein ε4 modulates phenotype of butyrylcholinesterase in CSF of patients with Alzheimer's disease. J Alzheimers Dis 28: 443-458. PubMed: 22012848. 22012848 10.3233/JAD-2011-111088

[31] Balabanova S, Richter HP, Antoniadis G, Homoki J, Kremmer N et al. (1984) 25-Hydroxyvitamin D, 24, 25-dihydroxyvitamin D and 1,25-dihydroxyvitamin D in human cerebrospinal fluid. Klin Wochenschr 62: 1086–1090. doi:10.1007/BF01711378. PubMed: 6334780. 6334780

[32] Holmøy T, Moen SM, Gundersen TA, Holick MF, Fainardi E et al. (2009) 25-hydroxyvitamin D in cerebrospinal fluid during relapse and remission of multiple sclerosis. Mult Scler 15: 1280-1285. doi:10.1177/1352458509107008. PubMed: 19808741. 19808741

[33] Holick MF, Binkley NC, Bischoff-Ferrari HA, Gordon CM, Hanley DA et al. (2011) Evaluation, treatment, and prevention of vitamin D deficiency: an Endocrine Society clinical practice guideline. J Clin Endocrinol Metab 96: 1911-1930. doi:10.1210/jc.2011-0385. PubMed: 21646368. 21646368

[34] Annweiler C, Fantino B, Schott AM, Krolak-Salmon P, Allali G et al. (2012) Vitamin D insufficiency and mild cognitive impairment: cross-sectional association. Eur J Neurol 19: 1023-1029. doi:10.1111/j.1468-1331.2012.03675.x. PubMed: 22339714. 22339714

[35] Atack JR, May C, Kaye JA, Kay AD, Rapoport SI (1988) Cerebrospinal fluid cholinesterases in aging and in dementia of the Alzheimer type. Ann Neurol 23: 161-167. doi:10.1002/ana.410230736. PubMed: 3377438. 3377438

[36] Appleyard ME, Smith AD, Berman P, Wilcock GK, Esiri MM et al. (1987) Cholinesterase activities in cerebrospinal fluid of patients with senile dementia of Alzheimer type. Brain 110: 1309-1322. doi:10.1093/brain/110.5.1309. PubMed: 3676702. 3676702

[37] Atack J (1989) Cerebrospinal fluid neurochemical markers in Alzheimer's disease. In Boller F Katzman R Rascol A Signoret JL Cristen Y, Biological markers of Alzheimer´s disease. Springer-Verlag Berlin pp. 1-16.

[38] Wallin A, Sjögren M, Blennow K, Davidsson P (2003) Decreased cerebrospinal fluid acetylcholinesterase in patients with subcortical ischemic vascular dementia. Dement Geriatr Cogn Disord 16: 200-207. doi:10.1159/000072803. PubMed: 14512714. 14512714

[39] Lehmann D, Johnston C, Smith A (1997) Synergy between the genes for butyrylcholinesterase K variant and apolipoprotein E4 in late-onset confirmed. Journal of Alzheimer'S Disease - Hum Mol Genet 6: 1933-1936. 10.1093/hmg/6.11.1933 9302273

[40] Kehoe PG, Williams H, Holmans P, Wilcock G, Cairns NJ et al. (1998) The butyrylcholinesterase K variant and susceptibility to Alzheimer's disease. J Med Genet 35: 1034-1035. doi:10.1136/jmg.35.12.1034. PubMed: 9863603. 9863603 PMC1051518

[41] McIlroy SP, Crawford VL, Dynan KB, McGleenon BM, Vahidassr MD et al. (2000) Butyrylcholinesterase K variant is genetically associated with late onset Alzheimer's disease in Northern Ireland. J Med Genet 37: 182-185. doi:10.1136/jmg.37.3.182. PubMed: 10699053. 10699053 PMC1734550

[42] Shah I, Petroczi A, Tabet N, Klugman A, Isaac M et al. (2012) Low 25OH vitamin D2 levels found in untreated Alzheimer's patients, compared to acetylcholinesterase-inhibitor treated and controls. Curr Alzheimer Res 9: 1069-1076. doi:10.2174/156720512803568975. PubMed: 22876849. 22876849

[43] Annweiler C, Fantino B, Gautier J, Beaudenon M, Thiery S et al. (2012) Cognitive effects of vitamin D supplementation in older outpatients visiting a memory clinic: a pre-post study. J Am Geriatr Soc 60: 793-795. doi:10.1111/j.1532-5415.2011.03877.x. PubMed: 22494292. 22494292

[44] Annweiler C, Herrmann FR, Fantino B, Brugg B, Beauchet O (2012) Effectiveness of the combination of memantine plus vitamin D on cognition in patients with Alzheimer disease: a pre-post pilot study. Cogn Behav Neurol 25: 121-127. doi:10.1097/WNN.0b013e31826df647. PubMed: 22960436. 22960436

